# A time-since-infection model for populations with two pathogens

**DOI:** 10.1016/j.tpb.2022.01.001

**Published:** 2022-01-17

**Authors:** Ferdinand Pfab, Roger M. Nisbet, Cheryl J. Briggs

**Affiliations:** Department of Ecology, Evolution and Marine Biology, University of California, Santa Barbara, USA

**Keywords:** Epidemics, Superinfection, Coinfection, Time-since-infection model, Kermack–McKendrick model, Partial differential equations

## Abstract

The pioneering work of Kermack and McKendrick (1927, 1932, 1933) is now most known for introducing the SIR model, which divides a population into discrete compartments for susceptible, infected and removed individuals. The SIR model is the archetype of widely used compartmental models for epidemics. It is sometimes forgotten, that Kermack and McKendrick introduced the SIR model as a special case of a more general framework. This general framework distinguishes individuals not only by whether they are susceptible, infected or removed, but additionally tracks the time passed since they got infected. Such time-since-infection models can mechanistically link within-host dynamics to the population level. This allows the models to account for more details of the disease dynamics, such as delays of infectiousness and symptoms during the onset of an infection. Details like this can be vital for interpreting epidemiological data.

The time-since-infection framework was originally formulated for a host population with a single pathogen. However, the interactions of multiple pathogens within hosts and within a population can be crucial for understanding the severity and spread of diseases. Current models for multiple pathogens mostly rely on compartmental models. While such models are relatively easy to set up, they do not have the same mechanistic underpinning as time-since-infection models. To approach this gap of connecting within-host dynamics of multiple pathogens to the population level, we here extend the time-since-infection framework of Kermack and McKendrick for two pathogens.

We derive formulas for the basic reproduction numbers in the system. Those numbers determine whether a pathogen can invade a population, potentially depending on whether the other pathogen is present or not. We then demonstrate use of the framework by setting up a simple within-host model that we connect to the population model. The example illustrates the context-specific information required for this type of model, and shows how the system can be simulated numerically. We verify that the formulas for the basic reproduction numbers correctly specify the invasibility conditions.

## 1. Introduction

Simultaneous infection of hosts with more than one pathogen is widespread. Many species are infected with multiple pathogens most of the time (Petney and Andrews, 1998).

Within a host, different pathogens can interact in several ways (Cox, 2001; Griffiths et al., 2011). They can facilitate each other when they suppress the host’s defense system such as in the case of pneumonia in HIV-infected humans (Walzer et al., 2008). Different pathogens can also repress each other, for example when they compete for resources within the host or boost similar defense systems such as in the case of cross-immunity between different influenza strains (Bhattacharyya et al., 2015). Interactions do not need to be symmetric, and there can be also no effect in one or both directions. In some instances interactions are direct, as for example macroparasites fighting physically for space in the host. In many other cases interactions are indirectly mitigated by the host’s state, e.g. its immune system and energy reserves.

Currently most models for the dynamics of multiple pathogens in a population are either agent based stochastic models (Ball, 2006) or, more commonly, extensions of the classic SIR framework (Ackleh and Allen, 2003; Bhunu et al., 2009; Alizon, 2013; Gao et al., 2016). SIR models are compartmental models that divide the population into discrete classes, i.e. for susceptible, infected and removed individuals. This allows to formulate such models with ordinary differential equations (ODEs), which are relatively easy to analyze and simulate. However these simple models assume all individuals within a compartment to have identical properties and thus do not allow for linking detailed within-host dynamics to the population level. An approach to addressing this link can be found in the groundbreaking series of papers by Kermack and McKendrick (1927, 1932, 1933). Although these papers are now most famous for introducing the simple ODE-based SIR model, they actually introduce that model as a special case of a general framework that tracks the distribution of individuals over a state space, such as the pathogen load and the state of the immune system. This does not only allow us to better capture empirical patterns such as the distribution of a latency period, but also defines mechanistic links from within-host dynamics to the population level (Breda et al., 2012). This approach is formulated with partial differential equations (PDEs). Kermack and McKendrick also show how such models can be simplified by tracking the time passed since a host was infected instead of tracking the pathogen load and the state of the immune system separately. This time-since-infection can then be used to derive host properties such as pathogen load, infectiousness and mortality. As written, such a time-since-infection model looks like an SI model because it does not need a compartment for removed (i.e. immune) individuals. However the PDE model is indeed the general case of an SIR model because removed individuals can still have a formal time-since-infection.

Here, we aim to fill the gap by connecting the dynamics of multiple pathogens within a host to the dynamics on the population level. We extend the time-since-infection PDE approach of Kermack and McKendrick for two pathogens, tracking the time-since-infection for each of them. Using general functional forms, we follow classical epidemiological theory to analyze the model. We derive the basic reproduction numbers, which determine whether a pathogen can invade a population with no pathogen present and/or into a population with the other pathogen at equilibrium. In Appendix A, we show an alternative way to determine invasibility of the pathogens. This approach requires more algebraic manipulations but bases on arguably more basic principles. Also, it can be additionally used to determine how fast a pathogen spreads in the early phase of an epidemic.

For applications, the within-host functions can be found either experimentally or with suitable sub-models, as in the works by Perelson and Weisbuch (1997) and Austin et al. (1998). To demonstrate the framework, we set up a simple sub-model for within-host pathogen dynamics and show how to connect it to the population model. We then simulate the system and compute the basic reproduction numbers. Finally we discuss applications and modifications for the modeling approach, and we list numerical methods that can be used to simulate such systems.

## 2. The single pathogen Kermack-McKendrick model

**Fig. 1.**
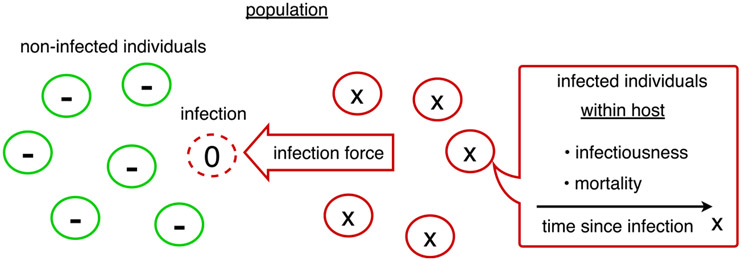
The original Kermack–McKendrick model describes the dynamics of a single pathogen in a population. It connects the within-host dynamics with the population level by tracking the time since infection *x* of infected individuals. At the time of contracting the pathogen, the time since infection is *x* = 0. The state of non-infected individuals is *x* = −.

The single pathogen model is schematized in Fig. 1. The model is described with a slightly different notation in the book by Perthame (2006).

### 2.1. Notation

In contrast to the formulation in the works of Perthame (2006) and Kermack and McKendrick (1927, 1932, 1933), we denote the density of infected and non-infected individuals with the same function, *n*(*t*, *x*). Here *t* is the current time, and *x* is the infection state. Non-infected hosts are characterized by *x* = −. For infected individuals the state is $x\in R_{\geq 0}$, representing the time passed since infection. This notation will simplify the formulation and the analysis of the models.

One consequence of this notation is that the function *n*(*t*, *x*) has different units depending on its argument. In particular, *n*(*t*, *x*) with $x\in R_{\geq 0}$ (infected individuals) has unit 1/*time* times the unit of *n*(*t*, −) (non-infected individuals), which is itself a density with, for example, unit 1/m^2^ for terrestrial organisms or 1/m^3^ for aquatic organisms. The difference in units can be seen when considering that the total population density is the sum of susceptible and infected individuals.


(1) $$n(t,\text{-})+\int_{0}^{\infty }n(t,x)dx$$

### 2.2. Model

We assume, that the population is well mixed, individuals with time-since-infection *x* ∈ *Ω* die at a rate *μ*(*x*), individuals with time-since-infection $x\in R_{\geq 0}$ (infected individuals) transmit the disease at rate *κ*(*x*), and new individuals are born into the population at rate *B* as non-infected. To keep the model simple, we do not track the age of individuals. We remark on this kind of additional population heterogeneities in the discussion. The components of the model are summarized in Table 1.

The rate of change of non-infected individuals is

(2) $$\frac{d}{dt}n(t,\;\text{-})=B-\mu (\text{-})n(t,\;\text{-})-\lambda (t)n(t,\;\text{-})$$

where λ is the infection force, defined by

(3) $$\lambda (t)=\int_{0}^{\infty }\kappa (x)n(t,x)dx$$

The change of individuals infected time $x\in R_{>0}$ ago is

(4) $$\frac{\partial }{\partial t}n(t,x)+\frac{\partial }{\partial x}n(t,x)+\mu (x)n(t,x)=0$$

The rate of new infections, which specifies the boundary condition for the PDE describing the dynamics of infected individuals is

(5) n(t,0)=λ(t)n(t,-)

We assume for the analysis that *B* > 0 and *μ*(−) > 0. This technically excludes the standard SIR model, which describes a closed population with no mortality of non-infected individuals and no new birth. In this special case of a closed population, the equilibria depend on the initial conditions. Taking this into account, our analysis could be readily extended for this case.

### 2.3. Equilibria

#### 2.3.1. Disease free equilibrium

We denote the disease free equilibrium by ${\bar{n}}_{0}$. At this equilibrium there are no infected individuals, ${\bar{n}}_{0}(x)=0$ for $x\in R_{\geq 0}$. The steady state of non-infected individuals is calculated by setting $\frac{d}{dt}n(t,\;\text{-})=0$,

(6) $${\bar{n}}_{0}(\text{-})=\frac{B}{\mu (\text{-})}$$

The disease free equilibrium is obviously stable due to the constant birth rate and the constant per capita mortality rate.

#### 2.3.2. Equilibrium with the pathogen

We denote the equilibrium with the pathogen present by $\bar{n}$. The equilibrium is found by setting $\frac{\partial }{\partial t}n(t,x)=0$ for $x\in R_{\geq 0}$ and $\frac{d}{dt}n(t,\;\text{-})=0$. From the first condition we find for $x\in R_{\geq 0}$

(7) $$\frac{d}{dx}\bar{n}(x)=-\mu (x)\bar{n}(x)$$

This reflects the fact that the probability for an individual to be still alive time *x* after getting infected is

(8) $$F(x)=e^{-\int_{0}^{x}\mu (y)dy}$$

Together with the boundary condition at *x* = 0, this leads to $\bar{n}(x)=\bar{n}(0)F(x)$

(9) $$\bar{n}(x)=\bar{n}(0)F(x)\;\;=\bar{\lambda }\bar{n}(\text{-})F(x)$$

where we use that according to the definition of the boundary condition $\bar{n}(0)=\bar{\lambda }\bar{n}(\text{-})$. The equilibrium infection force $\bar{\lambda }$ is

(10) $$\bar{\lambda }=\int_{0}^{\infty }\kappa (x)\bar{n}(x)dx\;\;=\bar{\lambda }\bar{n}(\text{-})\int_{0}^{\infty }\kappa (x)F(x)dx$$

Dividing both sides by $\bar{\lambda }$ leads to the density of non-infected individuals

(11) $$\bar{n}(\text{-})=\frac{1}{\int_{0}^{\infty }\kappa (x)F(x)dx}$$

We now set $\frac{d}{dt}n(t,\text{-})=0$ and find 0 = *B —* μ(−)ϋ (−) — *λ n* (−)

(12) $$0=B-\mu (\text{-})\bar{n}(\text{-})-\bar{\lambda }\bar{n}(\text{-})$$

that means

(13) $$\bar{\lambda }=\frac{B-\mu (\text{-})\bar{n}(\text{-})}{\bar{n}(\text{-})}$$

Finally, we can use the closed forms for $\bar{\lambda }$ and $\bar{n}(\text{-})$ to express the density of infected individuals $\bar{n}(x)$ with $x\in R_{\geq 0}$

(14) $$\bar{n}(x)=\left(B-\frac{\mu (\text{-})}{\int_{0}^{\infty }\kappa (x)F(x)dx}\right)F(x)$$

### 2.4. Basic reproduction number and invasibility

The pathogen can invade a population when infected individuals infect on average more than one individual in a population consisting of susceptibles only, that is when the basic reproduction number *R*_0_ > 1 (Diekmann et al., 2012). The basic reproduction number can be calculated as the integral of the product of the rate individuals with time-since-infection *x* infected other individuals in a fully susceptible population, $\kappa (x){\bar{n}}_{0}(\text{-})$, and the probability F(x) that infected individuals reach this time-since-infection

(15) $$R_{0}=\int_{0}^{\infty }\kappa (x){\bar{n}}_{0}(\text{-})F(x)dx$$

This endemic equilibrium is stable whenever *R*_0_ > 1, see Magal et al. (2010).

## 3. The two pathogen extension of the Kermack–McKendrick model

**Fig. 2.**
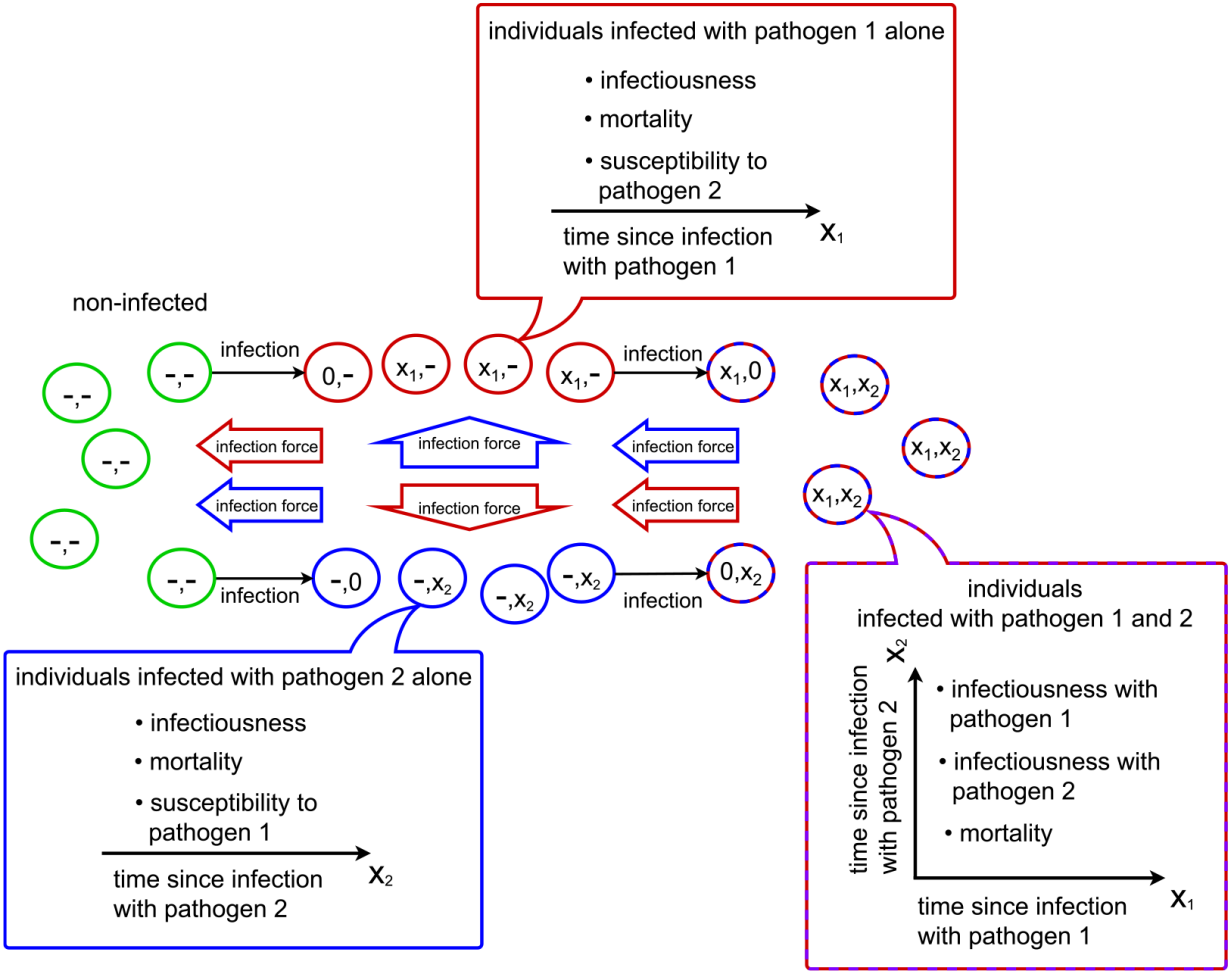
The extended Kermack–McKendrick model for the dynamics of two pathogens in a population. For both pathogens, the model tracks the time since infection, *x*_1_ and *x*_2_. At the time of contracting a pathogen, the corresponding time since infection starts at 0. For individuals not infected with a given pathogen, the corresponding state is “-”.

This model extends the single pathogen model for two pathogens as shown in Fig. 2. Hosts are characterized by whether/for how long they have been infected with either pathogen. We assume again that the population is well mixed. Additionally, we assume that individuals acquire only one pathogen at a time, even when getting in contact with a host that carries both pathogens.

### 3.1. Notation

The state variable *n*(*t*, *x*_1_, *x*_2_) is used to measure all types of individuals. The states *x*_1_ ∈ *Ω* and *x*_2_ ∈ *Ω* stand for the time-since-infection with pathogens 1 and 2, where $\Omega =\{\text{-}\}\cup R_{\geq 0}$. Individuals not infected with pathogen 1 and/or pathogen 2, have state *x*_1_ = − and/or *x*_2_ = −.

As before, the units of *n* depend on whether individuals are infected with pathogen 1 and/or 2. This can be demonstrated by noting that the total population density is

(16) $$n(t,\text{-},\text{-})+\int_{0}^{\infty }n(t,x_{1},\text{-})dx_{1}+\int_{0}^{\infty }n(t,\text{-},x_{2})dx_{2}\;+\int_{0}^{\infty }\int_{0}^{\infty }n(t,x_{1},x_{2})dx_{1}dx_{2}$$

### 3.2. Model

The model components of the two pathogen Kermack-McKendrick model are summarized in Table 2. The differential equation for non-infected individuals is

(17) $$\frac{d}{dt}n(t,\text{-},\text{-})=B-(\mu (\text{-},\text{-})+\beta_{1}(\text{-})\lambda_{1}(t)+\beta_{2}(\text{-})\lambda_{2}(t))n(t,\text{-},\text{-})$$

where the infection forces from pathogens 1 and 2 are respectively

(18) $$\lambda_{1}(t)=\int_{0}^{\infty }\left(\kappa_{1}(x_{1},\text{-})n(t,x_{1},\text{-})+\int_{0}^{\infty }\kappa_{1}(x_{1},x_{2})n(t,x_{1},x_{2})dx_{2}\right)\;\lambda_{2}(t)=\int_{0}^{\infty }\left(\kappa_{2}(\text{-},x_{2})n(t,\text{-},x_{2})+\int_{0}^{\infty }\kappa_{2}(x_{1},x_{2})n(t,x_{1},x_{2})dx_{1}\right)\;\;\times dx_{2}$$

The PDEs for single-infected individuals with pathogen 1 and pathogen 2 respectively are

(19) $$\frac{\partial }{\partial t}n(t,x_{1},\text{-})+\frac{\partial }{\partial x_{1}}n(t,x_{1},\text{-})+(\beta_{2}(x_{1})\lambda_{2}(t)+\mu (x_{1},\text{-}))n(t,x_{1},\text{-})\;\;=0\;\frac{\partial }{\partial t}n(t,\text{-},x_{2})+\frac{\partial }{\partial x_{2}}n(t,\text{-},x_{2})+(\beta_{1}(x_{2})\lambda_{1}(t)+\mu (\text{-},x_{2}))n(t,\text{-},x_{2})\;\;=0$$

and the PDE for co-infected individuals is

(20) $$\frac{\partial }{\partial t}n(t,x_{1},x_{2})+\frac{\partial }{\partial x_{1}}n(t,x_{1},x_{2})+\frac{\partial }{\partial x_{2}}n(t,x_{1},x_{2})\;\;+\mu (x_{1},x_{2})n(t,x_{1},x_{2})=0$$

The boundary conditions are

(21) $$\;n(t,\;0\;,\text{-})=\beta_{1}(\text{-})\lambda_{1}(t)n(t,\text{-},\text{-})\;\;n(t,\text{-},\;0)=\beta_{2}(\text{-})\lambda_{2}(t)n(t,\text{-},\text{-})\;n(t,\;0,x_{2})=\beta_{1}(x_{2})\lambda_{1}(t)n(t,\text{-},x_{2})\;n(t,x_{1},\;0)=\beta_{2}(x_{1})\lambda_{2}(t)n(t,x_{1},\text{-})$$

Again, we assume for the analysis that *B* > 0 and *μ*(−, −) > 0. The case that new birth and mortality of non-infected individuals are negligible, *B* = 0 and *μ*(−, −) = 0, can be analyzed similarly but we do not write out the arguments.

### 3.3. Equilibria

#### 3.3.1. Disease free state

We denote the equilibrium with no pathogen present by ${\bar{n}}_{0}$. Analogous to the model for only one pathogen, the density of non-infected individuals in the absence of both pathogens is

(22) $${\bar{n}}_{0}(\text{-},\text{-})=\frac{B}{\mu (\text{-},\text{-})}$$

#### 3.3.2. One pathogen alone

*Pathogen 1* We denote the equilibrium with only pathogen 1 present by ${\bar{n}}_{1}$. Analogously to the model for only one pathogen, the equilibrium of non-infected individuals is

(23) $${\bar{n}}_{1}(\text{-},\text{-})=\frac{1}{\beta_{1}(\text{-})\int_{0}^{\infty }\kappa_{1}(x_{1},\text{-})F_{1}(x_{1})dx_{1}}$$

where $F_{1}(x_{1})$ is the probability that an individual infected with pathogen 1 time *x*_1_ ago is still alive

(24) $$F_{1}(x_{1})=e^{-\int_{0}^{x_{1}}\mu (y_{1},\text{-})dy_{1}}$$

The equilibrium of individuals infected with pathogen 1 is

(25) $${\bar{n}}_{1}(x_{1},\text{-})={\bar{\lambda }}_{1,1}\beta (\text{-}){\bar{n}}_{1}(\text{-},\text{-})F_{1}(x_{1})$$

where the infection force for pathogen 1 is

(26) $${\bar{\lambda }}_{1,1}=\frac{B-\mu (\text{-},\text{-}){\bar{n}}_{1}(\text{-},\text{-})}{\beta (\text{-}){\bar{n}}_{1}(\text{-},\text{-})}$$

*Pathogen 2* In the same way, we denote the equilibrium with pathogen 2 alone by ${\bar{n}}_{2}$ and find

(27) $$\;{\bar{n}}_{2}(\text{-},\text{-})=\frac{1}{\beta_{2}(\text{-})\int_{0}^{\infty }\kappa_{2}(\text{-},x_{2})F_{2}(x_{2})dx_{2}}\;{\bar{n}}_{2}(\text{-},x_{2})={\bar{\lambda }}_{2,2}\beta (\text{-}){\bar{n}}_{2}(\text{-},\text{-})F_{2}(x_{1})$$

where $F_{2}(x_{2})$ is the probability that an individual infected time *x*_2_ ago is still alive

(28) $$F_{2}(x_{2})=e^{-\int_{0}^{x_{2}}\mu (\text{-},x_{2})dy_{1}}$$

and the infection force is

(29) $${\bar{\lambda }}_{2,2}=\frac{B-\mu (\text{-},\text{-}){\bar{n}}_{2}(\text{-},\text{-})}{\beta (\text{-}){\bar{n}}_{2}(\text{-},\text{-})}$$

#### 3.3.3 Both pathogens

The equilibrium with both pathogens can be expressed through an implicit set of equations. We do not need this for our analysis of the model, but for completeness the equations are stated in Appendix B.

### 3.4 Basic reproduction numbers and invasibility

#### 3.4.1 One pathogen alone

The criterion that determines whether each pathogen can invade into a disease-free host population can be calculated as in the single pathogen model.

**Pathogen 1** can invade a non-infected population at equilibrium if *R*_1,0_ > 1, where

(30) $$R_{1,0}=\int_{0}^{\infty }\kappa (x_{1},\text{-})\beta_{1}(\text{-}){\bar{n}}_{0}(\text{-})F_{1}(x_{1})dx_{1}$$

**Pathogen 2** can invade a non-infected population if *R*_0,2_ > 1, where

(31) $$R_{2,0}=\int_{0}^{\infty }\kappa (\text{-},x_{2})\beta_{2}(\text{-}){\bar{n}}_{0}(\text{-})F_{2}(x_{2})dx_{2}$$

#### 3.4.2 Invading a population with the other pathogen at equilibrium

Here we show how to find whether a second pathogen can invade into a population with a resident pathogen at equilibrium. As in the single pathogen case, this criterion is expressed by means of a basic reproduction number. The criterion we are about to derive makes sense only when the resident pathogen can persist in the population on its own (this condition is given through the basic reproduction numbers for single pathogens); otherwise the corresponding criterion would be the invasion into a disease free equilibrium, as derived before. Note also, that the equilibrium with a single pathogen (the resident pathogen) is stable (Magal et al., 2010), so indeed the equilibrium densities define the environment for the invading pathogen.

We apply an efficient method described in the book by Diekmann et al. (2012). In Appendix A we present an alternative method, which arguably requires less theoretical background at the cost of more additional algebraic manipulations. In contrast to the approach presented here, the alternative method also provides the speed with which the second pathogen spreads.

##### Theoretical background

Diekmann et al. (2012) describe how to calculate the basic reproduction number in structured population. This can be, for example, an age structure, or differences in contact rates within the population. In our case, the structure corresponds to the distribution of the time-since-infection with the resident pathogen (before the second pathogen arrives). Diekmann et al. (2012) cover three cases for the structure of the population: (1) discrete states, (2) continuous state, and (3) a mixture of discrete and continuous states. Our model corresponds to the third case, because the state of an individual, the time-since-infection, can be either the sign “-” indicating no infection, or for infected individuals a real number indicating the time passed since the infection. To understand the background of the method, it can be useful to start with the first two cases described by Diekmann et al. (2012). The procedure builds on the state space *Ω*, which contains all possible states for an individual at the time of getting infected. The density of individuals in any subset *ω* ⊆ *Ω* is written as *m*(*ω*). If *Ω* would be either discrete or continuous, *m* could be expressed as a sum or an integral; in our case *m* will be a combination of both. The next step is to find the expected number *Λ*(*η*)(*ω*) of individuals who get infected while in a state in *ω* by an individual that itself was in state *η* at the time of getting infected. This allows us to define the next-generation operator *K*, which gives the number and distribution of secondary cases given *m* and *ω*. Using the Lebesgue notation for integrals, this operator is given by

(32) $$\left(Km)(\omega )=\int_{\Omega }\Lambda (\eta )(\omega )m(d\eta \right)$$

The basic reproduction number *R*_0_ is the spectral radius of *K*. In words, the spectral radius is the average number of infections from a random infected individual when a stable state distribution is reached. Typically, *R*_0_ is the largest eigenvalue of *K*, and the corresponding eigenfunction *m* describes the stable state distribution. For details, see Diekmann et al. (2012). The pathogen can invade the population if and only if *R*_0_ > 1.

Generally, *R*_0_ has no analytic form and can be found only numerically. However, *R*_0_ has an analytic form when the expectation for the number of secondary infections can be expressed as a product with one of the factors depending only on *η* and the other only on *ω*,

(33) Λ(η)(ω)=α(ω)b(η)

This means that the state of the receiving individual does not depend on the state of the infecting individual. This is true for our model, because infectiousness is tunneled through the infection force. In this special case the basic reproduction number becomes

(34) $$R_{0}=\int_{\Omega }b(\eta )\alpha (d\eta )$$

##### Application

Here we find the reproduction number *R*_2,1_ for **pathogen 2 invading into a population where pathogen 1 is resident**. Pathogen 2 can invade if *R*_2,1_ > 1. At the beginning of the invasion, the population is at the equilibrium with only pathogen 1 present, given by ${\bar{n}}_{1}$. The state space is the time-since-infection with the resident pathogen, $\Omega =\{\text{-}\}\cup R_{\geq 0}$. To apply the theorem described above, we define the measure *m*(*ω*) as the total density of individuals with state in *ω* ∈ *Ω*

(35) $$m(\omega )=\delta_{\text{-}}(\omega ){\bar{n}}_{1}(-)+\int_{\omega ∕\{\text{-}\}}{\bar{n}}_{1}(x_{1},\text{-})dx_{1}$$

where *δ*_−_ is the unit mass at - (delta function).

We can express the expected number *Λ*(*η*)(*ω*) of cases (infections with pathogen 2) infected in state in *ω* ⊆ *Ω* (time-since-infection with pathogen 1) caused by an individual that was itself infected (with pathogen 2) while it was in state *η* ∈ *Ω* (time-since-infection with pathogen 1) as in Eq. (33). Here *α*(*ω*) is composed of susceptibility and density of individuals in state in *ω*

(36) $$\alpha (\omega )=\int_{\omega }\beta_{2}(\eta )m(d\eta )$$

and *b*(*η*) is the life time “infection force” from an individual that was infected while it was in state *η*. We assemble this function using a set of probabilities. We need the probability that an individual infected a time *x*_2_ ago with pathogen 2 while not infected with pathogen 1 is still alive and still not infected with pathogen 1

(37) $$F_{\text{-},2}(x_{2},\lambda_{1})=e^{-\int_{0}^{x_{2}}(\mu (\text{-},y_{2})+\lambda_{1}\beta_{1}(y_{2}))dy_{2}}$$

where *λ*_1_ is the infection force of pathogen 1. Additionally we need the survival probabilities of individuals infected with both pathogens. The survival probability from the time of getting infected with a second pathogen of an individual that was first infected with pathogen 1 a time *x*_1_ ago and then with pathogen 2 a time *x*_2_ ago is

(38) $$F_{1,2}(x_{1},x_{2})=e^{-\int_{0}^{x_{2}}\mu (x_{1}-x_{2}+\sigma ,\sigma )d\sigma }$$

In the same way, the survival probability from the time of getting infected with a second pathogen for an individual first infected with pathogen 2 a time *x*_2_ ago and then with pathogen 1 a time *x*_1_ ago is

(39) $$F_{2,1}(x_{1},x_{2})=e^{-\int_{0}^{x_{1}}\mu (\sigma ,x_{2}-x_{1}+\sigma )d\sigma }$$

We now can formulate the “infection forces”. For individuals infected a time $\eta \in R_{\geq 0}$ with pathogen 1 at the time of contracting pathogen 2,

(40) $$b(\eta )=\int_{0}^{\infty }F_{1,2}(\eta +x_{2},x_{2})\kappa_{2}(\eta +x_{2},x_{2})dx_{2}$$

and for individuals not infected with pathogen 1 when contracting pathogen 2,

(41) b(-)=∫0∞F-,2(x2,λ¯1,1)(κ2(-,x2)+λ¯1,1β1(x2))(×∫0∞κ2(x1,x1+x2)F2,1(x1,x2+x1)dx1)dx2

We can now state the basic reproduction number *R*_2,1_ using Eq. (34)

(42) R2,1=∫Ωb(η)α(dη)=b(-)a(-)+∫0∞b(η)a(η)dη=β2(-)n¯1(-,-)∫0∞F-,2(x2,λ¯1,1)(κ2(-,x2)+λ¯1,1β1(x2))(×∫0∞κ2(x1,x1+x2)F2,1(x1,x2+x1)dx1)dx2+∫0∞β2(η)n¯(η,-)∫0∞F1,2(η+x2,x2)κ2(η+x2,x2)dx2dη

Pathogen 2 is resident, pathogen 1 is invading

In the same way, the basic reproduction number for pathogen 1 invading a population with pathogen 2 at equilibrium is

(43) R1,2=β1(-)n¯1(-,-)∫0∞F1,-(x1,λ¯2,2)(κ1(x2,-)+λ¯2,2β2(x1))(×∫0∞κ1(x1+x2,x2)F1,2(x1+x2,x2)dx2)dx1+∫0∞β1(η)n¯2(-,η)∫0∞F2,1(x1,η+x1)κ1(x1,η+x1)dx1dη

whereby additionally

(44) $$F_{1,\text{-}}(x_{1},\lambda_{2})=e^{-\int_{0}^{x_{1}}(\mu (\text{-},y_{1})+\lambda_{2}\beta_{2}(y_{1}))dy_{1}}$$

is the probability that an individual infected a time *x*_1_ ago with pathogen 1 while not infected with pathogen 2 is still alive and still not infected with pathogen 2.

## 4 Within-host dynamics

This section introduces a simple within-host model that can be analyzed using our framework for the population dynamics. The within-host model is not intended to describe any specific real pathogens but it is presented as a minimal example that demonstrates how the population model works and precisely which within-host processes are required.

This within-host model describes the dynamics of the load of two pathogens within an individual, *p*_1_ and *p*_2_. Each pathogen is assumed to trigger an immune response, *q*_1_ and *q*_2_. Cross immunity can help individuals infected with one pathogen fight an infection with the other pathogen. Conversely, the pathogens can facilitate each other by repressing an immune response. The parameters for this model are summarized in Table 3. The table also offers sample values for the within-host parameters and for the other parameters required for the population model.

The pathogen dynamics are given by a baseline growth rate and the host immune responses so that

(45) $$\frac{dp_{i}(t)}{dt}=\left(r_{i}-\sum_{j\in \{1,2\}}c_{i,j}q_{j}(t)\right)p_{i}(t)$$

The dynamics of the immune responses are

(46) $$\frac{dq_{j}(t)}{dt}=\frac{p_{j}(t)}{1+\sum_{i\in \{1,2\}}h_{j,i}p_{i}(t)}$$

**Fig. 3.**
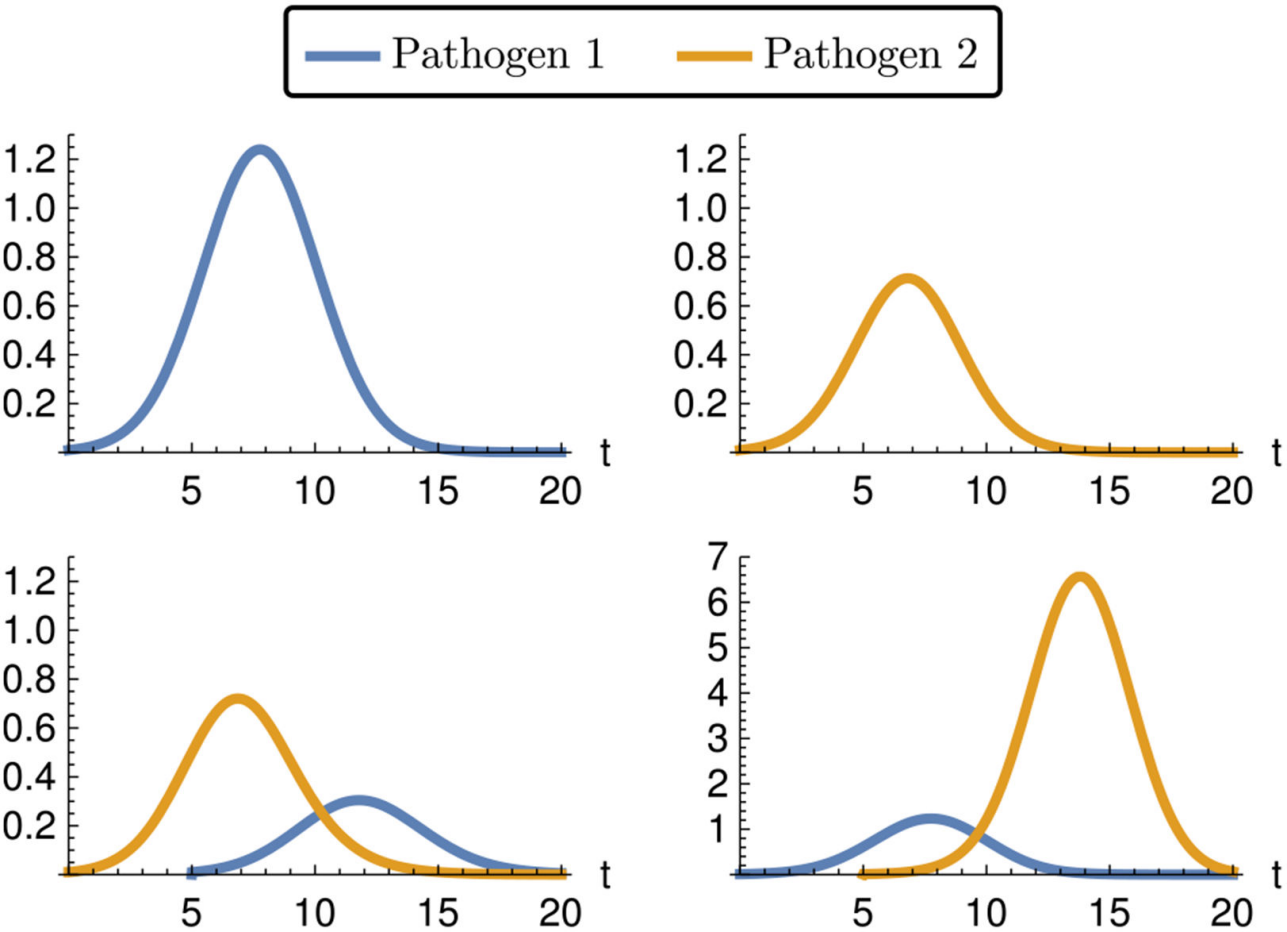
Example within-host dynamics. Clockwise from top left: Pathogen 1 introduced alone; pathogen 2 introduced alone; pathogen 1 introduced after pathogen 2; pathogen 2 introduced after pathogen 1. Pathogens are introduced at *t* = 0 and at *t* = 5 (for the two-pathogen scenarios). Pathogen 2 inhibits pathogen 1 due to a shared immune system, but pathogen 1 facilitates pathogen 2 because it represses the host’s reaction. Note the different axis scales. Parameter values are given in Table 3.

Infections are started with an initial pathogen load *z*_*i*_. The corresponding immune systems are at zero at this point. Simulations of the within-host model are shown in Fig. 3. The numerical example covers different cases for possible pathogen interactions. Pathogen 1 suppresses the immune response for pathogen 2 (facilitation), while the immune response triggered by pathogen 2 helps fight an infection with pathogen 1 (cross immunity).

The within-host processes are connected to the population level with the following functions. The infectiousness of the host with pathogen *i* is

(47) $$\kappa_{i}(t)=k_{i}p_{i}(t)$$

and the mortality rate of the host is

(48) $$\mu (t)=\mu_{0}+\sum_{i\in \{1,2\}}m_{i}p_{i}(t)$$

**Fig. 4.**
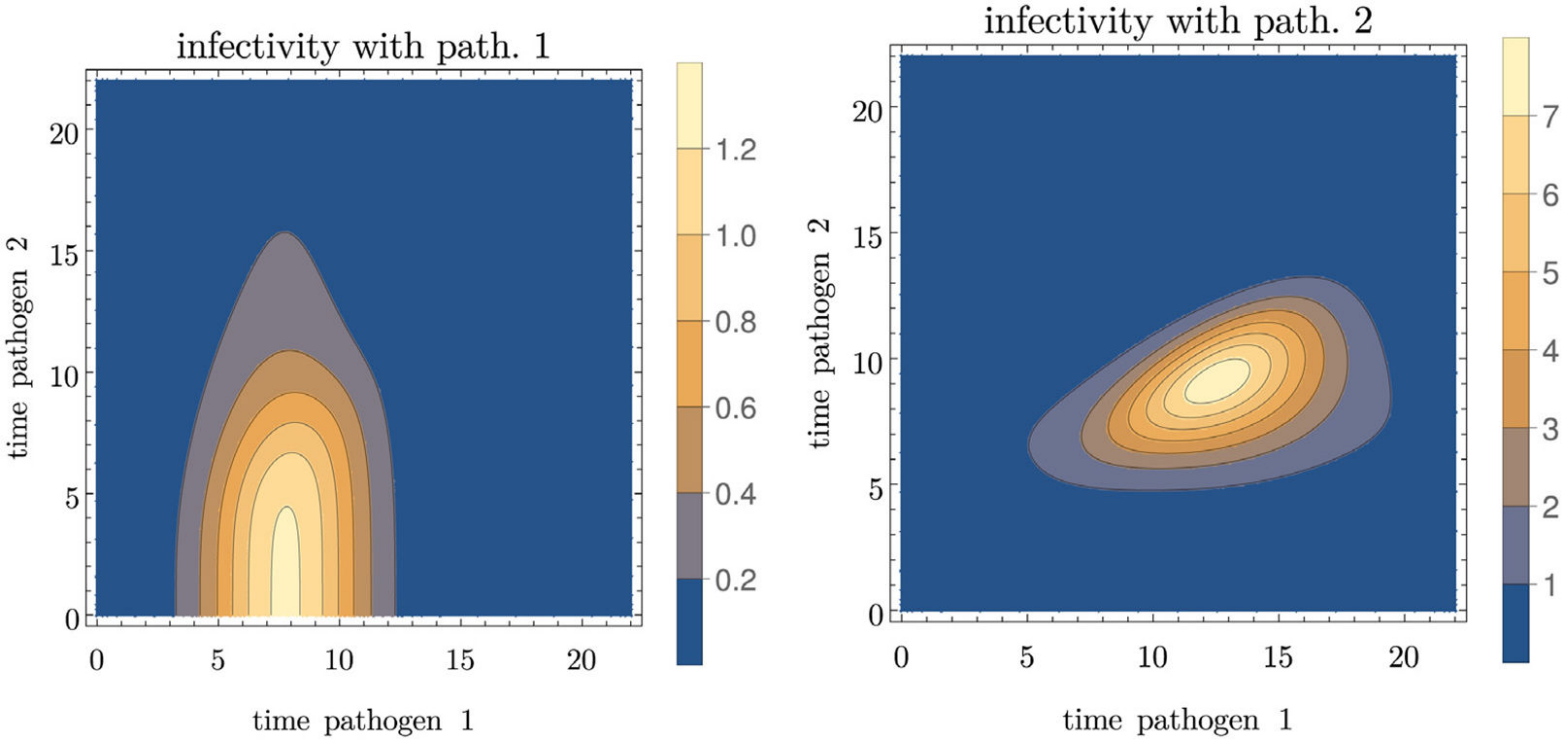
Example for the infectiousness with pathogen 1 (left) and pathogen 2 (right) as functions of the times since infection with the two pathogens. Parameter values are given in Table 3.

**Fig. 5.**
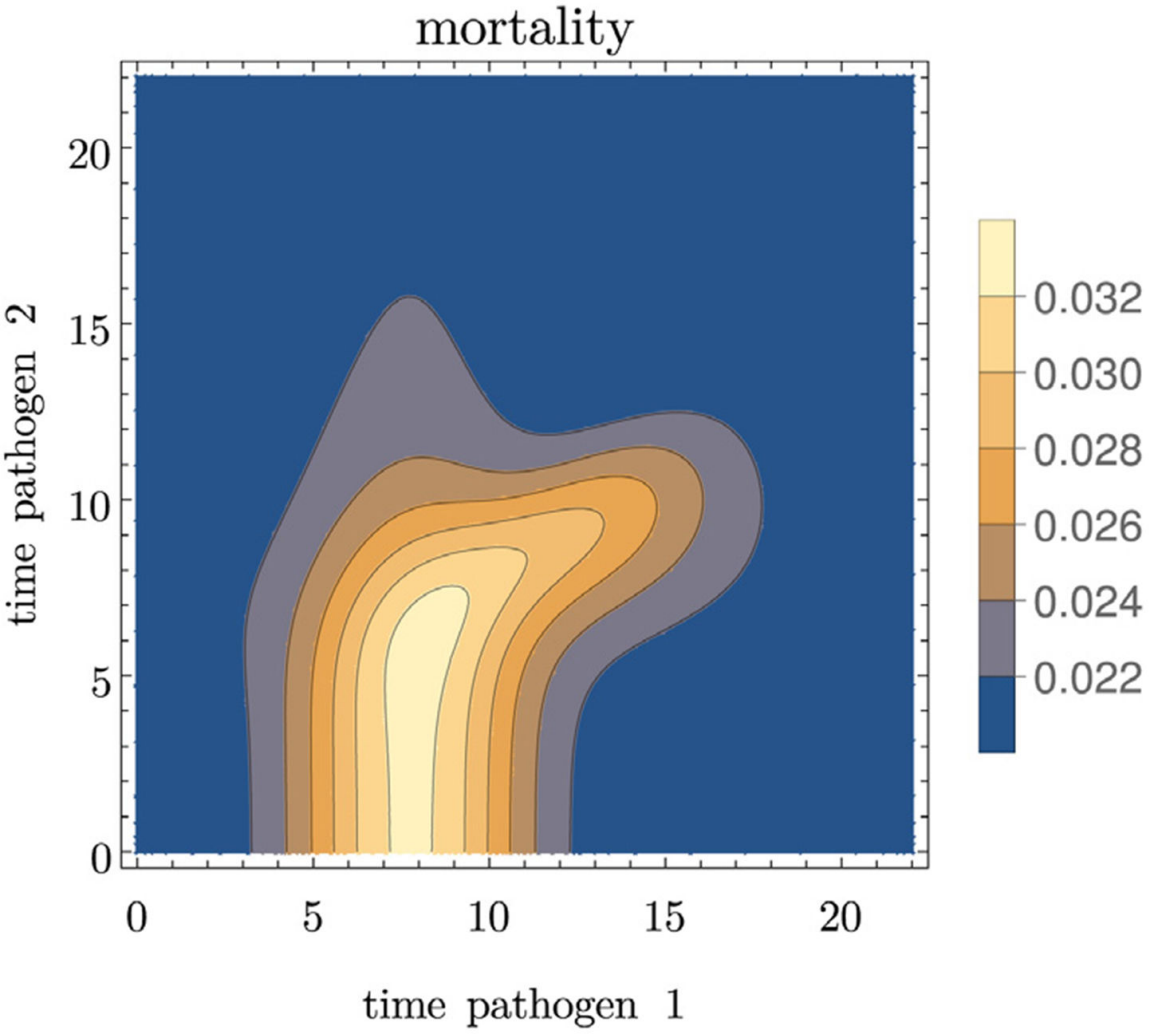
Example for mortality as a function of the time since infection with either pathogen. Parameter values are given in Table 3.

We then run within-host simulations with different times for introducing the two pathogens to compute the infectiousness and the mortality as functions of the times-since-infection with both pathogens, shown in Figs. 4 and 5. Those are the functions that feed into the population model.

Finally, the population model needs the susceptibility to either pathogen, given whether and for how long an individual has been infected with the other pathogen. We assume that the susceptibility to pathogen *i* depends on a baseline level, a cross immunity parameter, and the immune response to the other pathogen, *q*_*j*_(*t*), so that

(49) $$\beta_{i}(t)=b_{i}e^{-s_{i}q_{j}(t)}$$

Note that this is only relevant for individuals that have not been previously infected with the focal pathogen because individuals do not return to the uninfected class and cannot get reinfected with the same pathogen in the current form of the model.

**Fig. 6.**
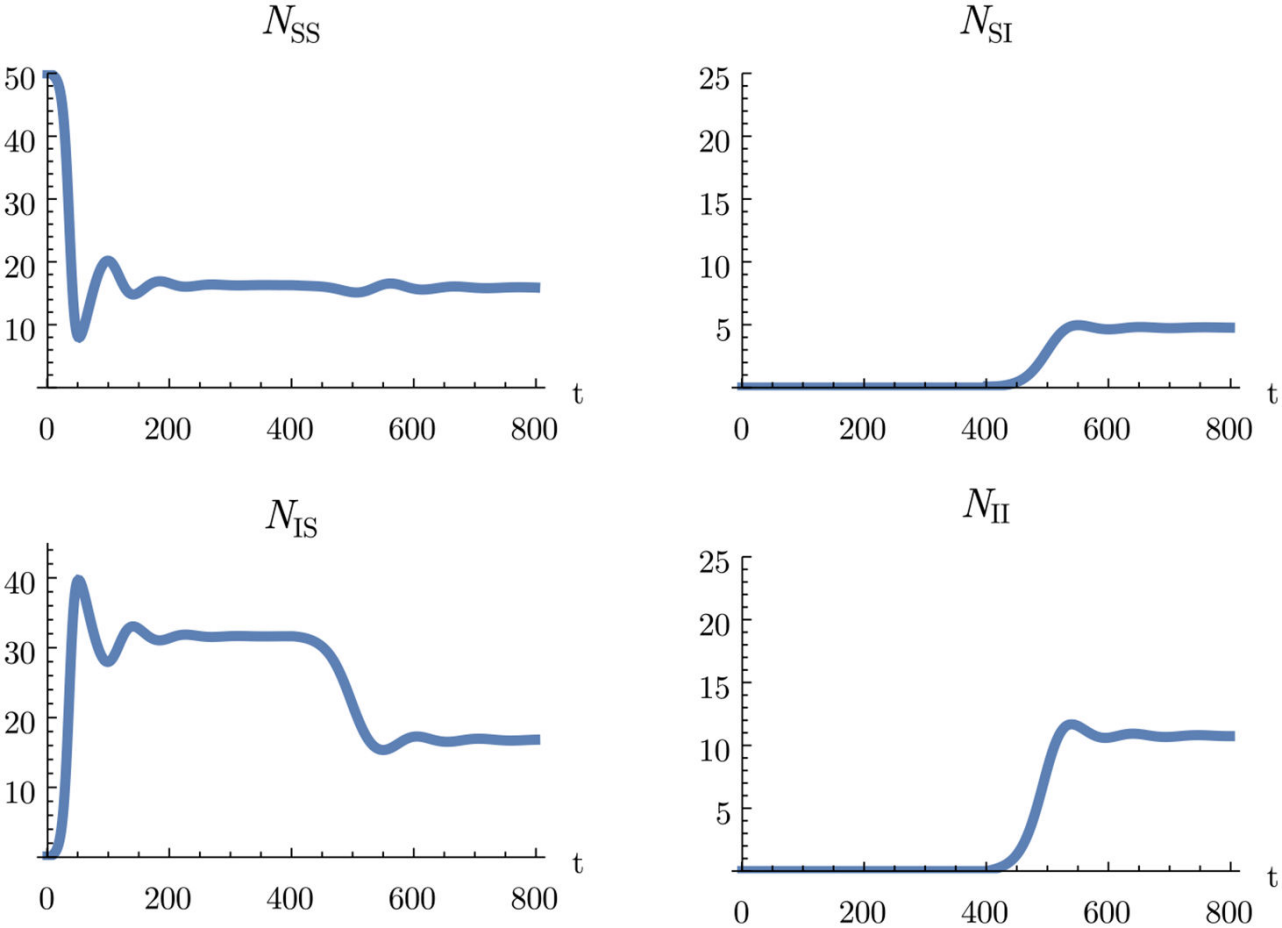
Simulations of the population dynamics. The within-host dynamics are specified by our example model. Individuals are binned together for the plots by whether they are infected with pathogen 1 and/or pathogen 2 (regardless of the time since infection). *N_SS_*: individuals not infected with either pathogen; *N*_*IS*_: individuals infected with only pathogen 1; *N_SI_* : individuals infected with only pathogen 2; *N_II_* : individuals infected with both pathogens. The system is started with the susceptible population at the disease-free equilibrium. Pathogen 1 is introduced at time *t* = 0, and pathogen 2 is introduced at time *t* = 400. Both pathogens are introduced by adding a small amount of individuals (at a density of 0.1) that are freshly infected with the pathogen to introduce and are not infected with the other pathogen. Parameter values are given in Table 3.

Now the within-host dynamics can be connected to the population model. Fig. 6 shows model runs, with the first pathogen being introduced at *t* = 0 and the second pathogen being introduced at *t* = 400. The plots show the individuals that have never been infected with either pathogen, *N*_*SS*_, individuals that have been only infected with pathogen 1 or 2, *N*_*IS*_ and *N*_*SI*_, and individuals that have been infected with both pathogens, *N*_*II*_. The classes for infected hosts lump together individuals regardless of how much time passed since their infection. They are defined by

(50) $$N_{SS}(t)=n(t,\text{-},\text{-})\;N_{IS}(t)=\int_{0}^{\infty }n(t,x_{1},\;\text{-})dx_{1}\;N_{SI}(t)=\int_{0}^{\infty }n(t,\text{-},x_{2})dx_{2}\;N_{II}(t)=\int_{0}^{\infty }\int_{0}^{\infty }n(t,x_{1},x_{2})dx_{1},dx_{2}$$

In the simulated population dynamics, pathogen 2 is mainly carried by individuals that have been already infected with pathogen 1 (*N*_*II*_ is larger than *N*_*SI*_). This reflects the within-host dynamics, where pathogen 2 is opportunistic and can grow best in individuals that have been recently infected with the other pathogen.

Numerically, the simulations are implemented in *Mathematica* (Wolfram Research, 2021) using a simplified version of the escalator boxcar train method (De Roos, 1988). For our purpose, this method tracks cohorts of individuals within given ranges for the time-since-infection with either pathogen.

**Fig. 7.**
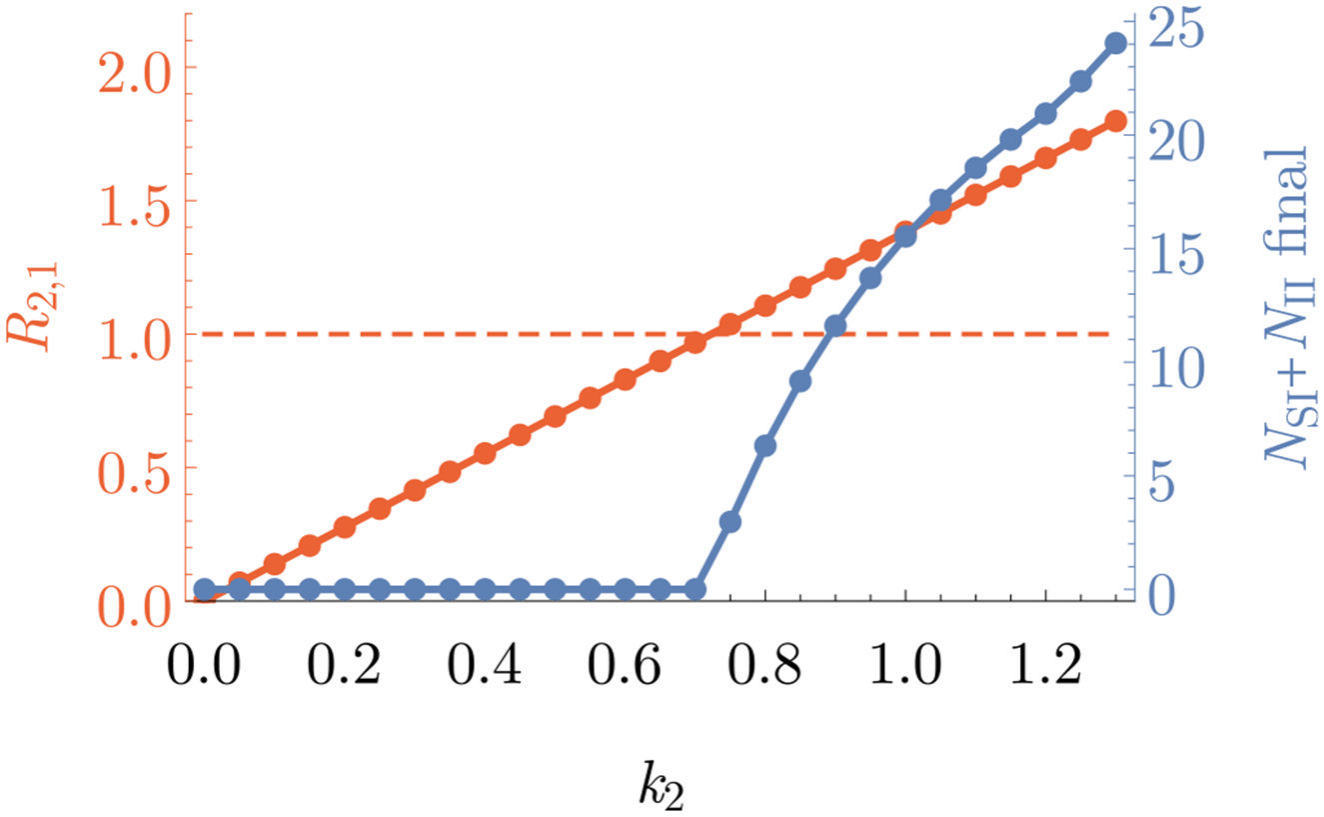
The basic reproduction number *R*_2,1_ and the final density of individual infected with pathogen 2 after a long simulation (at *t* = 2000) in dependence of the infectiousness of pathogen 2, *k*_2_. The within-host dynamics are specified by our example model. Pathogen 1 is introduced at the beginning of the simulation and pathogen 2 is introduced at *t* = 400. The parameter values are (except for *k*_2_) the same as in Fig. 6. The plot shows that pathogen 2 can only get established in a population with pathogen 1 present when the corresponding basic reproduction number *R*_2,1_ > 1.

The basic reproduction numbers of the system can be computed as described. The values for our numerical example are given in Table 4. In our example, the basic reproduction number *R*_1,2_ for pathogen 1 invading into a population with pathogen 2 present is not defined because pathogen 2 cannot persist in the population on its own. To check the analytical expression for the reproduction number in the two-pathogen model, we varied one of the parameters (the infectiousness of pathogen 2, *k*_2_) and verified that pathogen 2 can only get established in a population with pathogen 1 present (i.e. the density of individuals infected with pathogen 2 does not approach zero) when the basic reproduction number *R*_2,1_ > 1, see Fig. 7.

The code for the simulations and the calculations for the basic reproduction numbers is available freely online.^1^

## 5 Discussion

We formulated a general framework for the dynamics of two pathogens in a population. The time-since-infection approach allows for the connection of within-host dynamics to the population level by tracking the progression of the infection within hosts. We analyzed the framework by finding the basic reproduction numbers which determine whether each pathogen can invade a fully susceptible population and/or a population with the other pathogen present. To demonstrate the framework we set up an example for the within-host dynamics and linked it to the population level. The example shows how to derive the within-host functions from a model. In future applications, within-host functions can be either derived from experiments or from context-specific sub-models.

The framework can be easily generalized to different scenarios such as populations with arbitrarily many pathogens, other demographic features (including an explicit age structure or birth rates depending on the state of the population) or spatial structure. The current model assumes that infections are strictly sequential, but it would be relatively straight forward to generalize the model so that both pathogens can be transmitted together at a single contact event between an individual infected with both pathogens and a fully susceptible individual. The time-since-infection approach does usually assume that individuals can be infected once and that a re-exposure to the same pathogen will not change the course of the infection. Individuals that are no longer infectious still have a formal time-since-infection and are immune to the pathogen. Reinfections can be easily implemented when assuming that infected individuals can completely lose their infection and their “memory” of it (i.e. their immune system reverts to its original state). In this case one can simply let individuals become susceptible again at some rate that can depend on the time since infection. However, when the effect of reinfections depends on previous infections with the same pathogen (for example because the host’s immune system has some memory or because reinfections can boost an ongoing infection), one in principle needs to track the time since each (re-)infection. Depending on the application, it can in this case become easier to track the pathogen load and the state of the immune system directly in a PDE framework, rather than the time-since-infection.

Numerically the PDEs can be simulated with various methods such as the naive Euler scheme, which effectively discretizes the time and state space and computes the trajectories iteratively. The technique we implemented for our example is a modified version of the escalator boxcar train method (De Roos, 1988), which for our application follows cohorts of individuals acquiring an infection (or superinfection) during a certain time span. Yet another efficient method for simulating the model is to use the linear chain trick (Hurtado and Kirosingh, 2018; Diekmann et al., 2020), which is suitable when the time-since-infection functions for infectiousness, mortality and susceptibility can be assumed to have certain mathematical forms (such as the gamma distribution). All approaches are vulnerable to the “curse of dimensionality” that can slow down computations especially when the state space is large (e.g because of additional pathogens or other kinds of heterogeneity in the population). In many cases it can be practicable to reduce the state space by assuming that the state of an individual does not change after a certain time since infection (e.g. because it is no longer infectious or because the infection became chronic). Sometimes it is reasonable to assume that within-host dynamics are fast compared to the dynamics at the population level. In this case one can use equilibrium values for the within-host states, which reduces such systems to relative simple sets of ODEs (Gilchrist and Coombs, 2006; Coombs et al., 2007; Boldin and Diekmann, 2008).

Current research is revealing new insights on within-host dynamics of different pathogens and the interactions of individuals in population. The presented framework demonstrates how such within-host dynamics can be directly linked to the population level. We hope that this model approach will help to combine these levels and improve our understanding of how pathogens spread in populations.

## Figures and Tables

**Table 1:** Components of the single pathogen Kermack–McKendrick model.

Symbol	Description
*t*	Time
*x*	Time-since-infection $x\in \{\text{-}\}\cup R_{\geq 0}$ *x* = − → not infected $x\in R_{\geq 0}$ → infected at time *t* − *x*
*n*(*t, x*)	Density of individuals at time *t* infected time *x* ago
*μ*(*x*)	Mortality rate with time since infection *x*
*κ*(*x*)	Infectiousness with time since infection *x*
*λ*(*t*)	Infection force at time *t*
*B*	Birth rate (new non-infected individuals)

**Table 2:** Components of the two–pathogen Kermack–McKendrick model.

Symbol	Description
*x*_1_, *x*_2_	Time since infection with pathogens 1 and 2 *x*_1_, $x_{2}\in \{\text{-}\}\cup R_{\geq 0}$ *x*_1_ = − → not infected with pathogen 1 *x*_2_ = − → not infected with pathogen 2 $x_{1}\in R_{\geq 0}$ → infected with pathogen 1 at time *t* − *x*_1_ $x_{2}\in R_{\geq 0}$ → infected with pathogen 2 at time *t* − *x*_2_
*n*(*t*, *x*_1_, *x*_2_)	Individuals with times since infection *x*_1_ and *x*_2_ at time *t*
*μ*(*x*_1_, *x*_2_)	Mortality
*β*_1_(*x*_2_)	Susceptibility to pathogen 1
*β*_2_(*x*_1_)	Susceptibility to pathogen 2
*κ*_1_(*x*_1_, *x*_2_)	Infectiousness of pathogen 1
*κ*_2_(*x*_1_, *x*_2_)	Infectiousness of pathogen 2
*B*	Birth rate (non-infected individuals)

**Table 3:** Parameters for the within-host model and for connecting it to the population level.

Parameter	Description	Example
*r_i_*	Growth rate of pathogen *i*	*r*_1_ = 1.0 *r*_2_ = 1.0
*c_i,j_*	Effect of immune response *j* on pathogen *i*	*c*_1,1_ = 2 *c*_1,2_ = 0.5 *c*_2,1_ = 0 *c*_2,2_ = 2.5
*h*_*j,i*_	Inhibition of immune response *j* by pathogen *i*	*h*_1,1_ = 10 *h*_1,2_ = 0 *h*_2,1_ = 12 *h*_2,2_ = 10
*m_i_*	Mortality due to pathogen *i*	*m*_1_ = 0.01 *m*_2_ = 0.001
*k_i_*	Infectiousness of pathogen *i*	*k*_1_ = 1.0 *k*_2_ = 1.0
*b_i_*	Baseline susceptibility to pathogen *i*	*b*_1_ = 0.01 *b*_2_ = 0.005
*s_i_*	Reduction of susceptibility to pathogen *i* due to infection by the other pathogen	*s*_1_ = 0.5 *s*_2_ = 0
*z_i_*	Pathogen load to start infection with pathogen *i*	*z*_1_ = 0.01 *z*_2_ = 0.01
*μ*_0_	Mortality of non-infected individuals	*μ*_0_ = 0.02
*B*	Birth rate (non-infected individuals)	*B* = 1

**Table 4:** The basic reproduction numbers in our numerical example.

Symbol	Value	Description
*R*_1,0_	3.0	path. 1 invading a naive population
*R*_2,0_	0.8	path. 2 invading a naive population
*R*_1,2_	N/A because *R*_2,0_ < 1	path. 1 invading a population with path. 2
*R*_2,1_	1.4	path. 1 invading a population with path. 2

## Footnotes

1 https://github.com/ferdi-p/two-pathogen-time-since-infection-model.

## Appendix A. Alternative way to find the invasibility criteria

Here we describe an alternative way to find the threshold for whether a pathogen can invade in a population. The idea is to calculate the change of the infection force at the beginning of an outbreak, when the number of non-infected individuals can still be assumed to be at the disease free equilibrium. This also reveals the rate of propagation of a pathogen at an early stage of an epidemic.

### A.1. The single pathogen model

Following Gurney and Nisbet (1998) (section “Modelling age-structured populations in continuous time”), we use that during the invasion the population reaches a stable time-since-infection distribution and the infection force changes exponentially with a growth rate *ρ*

(A.1)
$$\frac{d}{dt}\lambda (t)=\rho \lambda (t)$$

This implies

(A.2)
$$\lambda (t+z)=\lambda (t)e^{\rho z}$$

Now using the definition of *λ*(*t*)

(A.3)
$$\frac{d}{dt}\lambda (t)=\frac{d}{dt}\int_{0}^{\infty }\kappa (x)n(t,x)dx\;\;=\frac{d}{dt}\int_{0}^{\infty }\kappa (x)F(x)\lambda (t-x){\bar{n}}_{0}(\text{-})dx\;\;=\frac{d}{dt}\lambda (t)\int_{0}^{\infty }\kappa (x)F(x)e^{-\rho x}{\bar{n}}_{0}(\text{-})dx$$

It follows

(A.4)
$$1=\int_{0}^{\infty }\kappa (x)F(x)e^{-\rho x}{\bar{n}}_{0}(\text{-})dx$$

The pathogen can invade if and only if *ρ* > 0. Since the right hand side of the equation is decreasing with *ρ*, this means

(A.5)
$$1<\int_{0}^{\infty }\kappa (x)F(x){\bar{n}}_{0}(\text{-})dx$$

The invasion threshold is equivalent to the criterion derived in the main text,

(A.6)
$$1<R_{0}$$

For large populations, *ρ* is the rate at which the pathogen is invading a susceptible population at an early stage of an out-break; i.e. when the density of susceptible individuals can still be assumed to be constant, but late enough that a stable time-since-infection distribution is reached.

### A.2. The two pathogen model

#### A.2.1. One pathogen alone

The criteria for whether each pathogen can invade into a disease-free host population can be found as in the single pathogen model.

#### A.2.2. Invading a population with the other pathogen at equilibrium

Pathogen 2 invading into a population where pathogen 1 is resident

Pathogen 2 can invade if *ρ* > 0, where

(A.7)
$$\frac{d\lambda_{2}(t)}{dt}=\rho \lambda_{2}(t)$$

The densities of individuals not-infected and infected with pathogen 1 alone, as well as the infection force of pathogen 1 are still given through the equilibrium with pathogen 1 alone, ${\bar{n}}_{1}$ and ${\bar{\lambda }}_{1,1}$.

We have

(A.8)
$$n(t,\text{-},x_{2})=\lambda_{2}(t-x_{2})\beta (0){\bar{n}}_{1}(\text{-},\text{-})F_{\text{-},2}(x_{2},{\bar{\lambda }}_{1,1})$$

We split up the individuals infected with both pathogens *n*(*t*, *x*_1_, *x*_2_) by the order of infection. Individuals first infected with pathogen 1 are denoted as *n*^12^ and individuals first infected with pathogen are denoted as *n*^21^. Using the definitions from the main text, we find

(A.9)
$$n^{12}(t,x_{1},x_{2})={\bar{n}}_{1}(x_{1}-x_{2},\text{-})\lambda_{2}(t-x_{2})\beta_{2}(x_{1}-x_{2})F_{1,2}(x_{1},x_{2})$$

and

(A.10)
$$n^{21}(t,x_{1},x_{2})=n(\text{-},x_{2}-x_{1},t){\bar{\lambda }}_{1,1}\beta_{1}(x_{2}-x_{1})F_{2,1}(x_{1},x_{2})\;\;=\lambda_{2}(t-x_{2})\beta (\text{-}){\bar{n}}_{1}(\text{-},\text{-})F_{\text{-},2}(x_{2}-x_{1},{\bar{\lambda }}_{1,1}){\bar{\lambda }}_{1,1}\;\;\times \beta_{1}(x_{2}-x_{1})F_{2,1}(x_{1},x_{2})$$

Now we use that for *x*_1_, $x_{2}\in R_{\geq 0}$ we have *n*(*t*, *x*_1_, *x*_2_) = *n*^12^(*t*, *x*_1_, *x*_2_) + *n*^21^(*t*, *x*_1_, *x*_2_). This leads to

(A.11)
ddtλ2(t)=ddt∫0∞(κ2(0,x2)n(t,-,-,x2))+(∫0∞κ2(x1,x2)n(t,x1,x2)dx1)dx2=ddt∫0∞(κ2(0,x2)n(t,-,x2))+∫x2∞κ2(x1,x2)n12(t,x1,x2)dx1+(∫0x2κ2(x1,x2)n21(t,x1,x2)dx1)dx2=ddt∫0∞(κ2(0,x2)n(t,-,x2))+∫x2∞κ2(x1,x2)n12(t,x1,x2)dx1+(∫0x2κ2(x1,x2)n21(t,x1,x2)dx1)dx2=ddt∫0∞(κ2(0,x2)λ2(t−x2)n¯1(-,-)F-,2(x2,λ¯1,1)+∫x2∞κ2(x1,x2)n¯(x1−x2,-)λ2(t−x2)β2(x1−x2)×F1,2(x1,x2)dx1+∫0x2κ2(x1,x2)λ2(t−x2)β(-)n¯1(-,-)×F-,2(x2−x1,λ¯1,1)λ¯1,1β1(x2−x1)(×F2,1(x1,x2)dx1)dx2

Using

(A.12)
$$\lambda_{2}(t-x)=\lambda_{2}(t)e^{-\rho x}$$

we have

(A.13)
1=∫0∞(κ2(0,x2)e−ρx2β2(-)n¯1(-,-)F-,2(x2,λ¯1,1))+∫x2∞κ2(x1,x2)n¯1(x1−x2,-)e−ρx2β2(x1−x2)×F1,2(x1,x2)dx1+∫0x2κ2(x1,x2)e−ρ(x2−x1)β2(-)n¯1(-,-)F-,2(x2−x1,λ¯1,1)(×λ¯1,1β1(x2−x1)F2,1(x1,x2)dx1)dx2

The right hand side is decreasing with *ρ*. That means *ρ* > 0 (and pathogen 2 is invading) when

(A.14)
1<∫0∞(κ2(0,x2)β2(-)n¯1(-,-)F-,2(x2,λ¯1,1))+∫x2∞κ2(x1,x2)n¯1(x1−x2,-)β2(x1−x2)F1,2(x1,x2)dx1+∫0x2κ2(x1,x2)β2(-)n¯1(-,-)F-,2(x2−x1,λ¯1,1)λ¯1,1(×β1(x2−x1)F2,1(x1,x2)dx1)dx2

Some simple manipulations of the integral limits show that the right hand side is equivalent to the basic reproduction number *R*_1,2_ we found before. Again the *ρ* is the rate at which the pathogen invades a large population at an early stage of the invasion.

Pathogen 1 invading into a population where pathogen 2 is resident

The invasibility criterion for this situation can be found analogously.

## Appendix B. Equilibrium with both pathogens

We derive an implicit formula for the equilibrium ${\bar{n}}_{12}$ with both pathogens. In this section we choose *x*_1_ and *x*_2_ to be in $R_{\geq 0}$. We use the notations defined in the main text. Setting the time derivatives to zero, we find the following relations.

(B.1)
$$\;\frac{dn(t,\text{-},\text{-})}{dt}=0\;{\bar{n}}_{12}(\text{-},\text{-})=\frac{B}{\mu (0,0)+\beta_{1}(\text{-}){\bar{\lambda }}_{1,12}+\beta_{2}(\text{-}){\bar{\lambda }}_{2,12}}\;\;\frac{\partial n(t,x_{1},\text{-})}{\partial t}=0\;\text{and}\;\frac{\partial n(t,\text{-},x_{2})}{\partial t}=0$$

Together with the boundary conditions *n*(*t*, 0, −) and *n*(*t*, −, 0), we find

(B.2)
$${\bar{n}}_{12}(x_{1},\text{-})={\bar{\lambda }}_{1,12}{\bar{n}}_{12}(\text{-},\text{-})F_{1,\text{-}}(x_{1},{\bar{\lambda }}_{2,12})\;{\bar{n}}_{12}(\text{-},x_{2})={\bar{\lambda }}_{2,12}{\bar{n}}_{12}(\text{-},\text{-})F_{\text{-},2}(x_{2},{\bar{\lambda }}_{1,12})$$

Using the boundary conditions for individuals infected with both pathogens, we find the following. The first and second terms of the sum correspond to individuals first infected with pathogen 1 and 2 respectively. All functions with negative argument are 0.

(B.3)
n¯12(x1,x2)=λ¯2,12β2(x1−x2)n¯12(x1−x2,-)F1,2(x1,x2)+λ¯1,12β1(x2−x1)n¯12(-,x2−x1)F2,1(x1,x2)=λ¯1,12λ¯2,12n¯12(-,-)(β2(x1−x2)F1,-(x1−x2,λ¯2,12))×F1,2(x1,x2)+β1(x2−x1)F-,2(x2−x1,λ¯1,12)(×F2,1(x1,x2))

The infection forces are given implicitly through

(B.4)
λ¯1,12=∫0∞(κ1(x1,-)n¯12(t,x1,-)(+∫0∞κ1(x1,x2)n¯12(t,x1,x2)dx2)dx1λ¯2,12=∫0∞(κ2(-,x2)n¯12(t,-,x2)(+∫0∞κ2(x1,x2)n¯12(t,x1,x2)dx1)dx2
